# Heterologous Prime-Boost Regimens with a Recombinant Chimpanzee Adenoviral Vector and Adjuvanted F4 Protein Elicit Polyfunctional HIV-1-Specific T-Cell Responses in Macaques

**DOI:** 10.1371/journal.pone.0122835

**Published:** 2015-04-09

**Authors:** Clarisse Lorin, Yannick Vanloubbeeck, Sébastien Baudart, Michaël Ska, Babak Bayat, Geoffroy Brauers, Géraldine Clarinval, Marie-Noëlle Donner, Martine Marchand, Marguerite Koutsoukos, Pascal Mettens, Joe Cohen, Gerald Voss

**Affiliations:** GSK Vaccines, Rixensart, Belgium; Commissariat a l'Energie Atomique(cea), FRANCE

## Abstract

HIV-1-specific CD4^+^ and CD8^+^ T lymphocytes are important for HIV-1 replication control. F4/AS01 consists of F4 recombinant fusion protein (containing clade B Gag/p24, Pol/RT, Nef and Gag/p17) formulated in AS01 Adjuvant System, and was shown to induce F4-specific polyfunctional CD4^+^ T-cell responses in humans. While replication-incompetent recombinant HIV-1/SIV antigen-expressing human adenoviral vectors can elicit high-frequency antigen-specific CD8^+^ T-cell responses, their use is hampered by widespread pre-existing immunity to human serotypes. Non-human adenovirus serotypes associated with lower prevalence may offer an alternative strategy. We evaluated the immunogenicity of AdC7-GRN (‘A’), a recombinant chimpanzee adenovirus type 7 vector expressing clade B Gag, RT and Nef, and F4/AS01 (‘P’), when delivered intramuscularly in homologous (PP or AA) and heterologous (AAPP or PPAA) prime-boost regimens, in macaques and mice. Vaccine-induced HIV-1-antigen-specific T cells in peripheral blood (macaques), liver, spleen, and intestinal and genital mucosa (mice) were characterized by intracellular cytokine staining. Vaccine-specific IgG antibodies (macaques) were detected using ELISA. In macaques, only the heterologous prime-boost regimens induced polyfunctional, persistent and balanced CD4^+^ and CD8^+^ T-cell responses specific to each HIV-1 vaccine antigen. AdC7-GRN priming increased the polyfunctionality of F4/AS01-induced CD4^+^ T cells. Approximately 50% of AdC7-GRN-induced memory CD8^+^ T cells exhibited an effector-memory phenotype. HIV-1-specific antibodies were detected with each regimen. In mice, antigen-specific CD4^+^ and CD8^+^ T-cell responses were detected in the mucosal and systemic anatomical compartments assessed. When administered in heterologous prime-boost regimens, AdC7-GRN and F4/AS01 candidate vaccines acted complementarily in inducing potent and persistent peripheral blood HIV-1-specific CD4^+^ and CD8^+^ T-cell responses and antibodies in macaques. Besides, adenoviral vector priming modulated the cytokine-expression profile of the protein-induced CD4^+^ T cells. Each regimen induced HIV-1-specific T-cell responses in systemic/local tissues in mice. This suggests that prime-boost regimens combining adjuvanted protein and low-seroprevalent chimpanzee adenoviral vectors represent an attractive vaccination strategy for clinical evaluation.

## Introduction

Evidence suggests that CD4^+^ and CD8^+^ T lymphocytes play a critical role in controlling human immunodeficiency virus type 1 (HIV-1) and simian immunodeficiency virus (SIV) replication. The appearance of virus-specific CD8^+^ T cells is closely associated with the initial drop in viremia occurring during primary HIV-1 infection [1–3], and vaccine-induced effector memory T-cell responses were shown to control pathogenic SIVmac239 replication in rhesus macaques, with some evidence of viral clearance [4,5]. In addition, there appears to be an inverse relationship between HIV-1-specific CD4^+^ T-cell functions and viral load [6]. In particular, CD4^+^ T cells have been shown to be implicated in the maintenance of functional memory CD8^+^ T cells [7,8]. The quality of HIV-1-specific T-cell responses seems to be crucial. Indeed, studies in long-term non-progressors and HIV controllers revealed that the presence of specific, polyfunctional CD4^+^ and CD8^+^ T cells in HIV-infected patients is associated with long-term non-progressing disease and low viral load [9–13]. While the ultimate objective of vaccine development efforts is the generation of a preventive HIV-1 vaccine inducing sterilizing immunity based on protective antibodies, a vaccine that is able to induce potent and polyfunctional T cell-mediated immune responses may also be beneficial in controlling viral replication in the early stages of infection (reviewed in [14,15]).

Human adenoviral vector-based vaccines expressing HIV-1 or SIV antigens have been shown to induce potent HIV-1 or SIV-specific T-cell responses in the periphery and at mucosal sites [16–23]. However, vaccination regimens using a replication-defective adenovirus serotype 5 vector (Ad5), alone or in prime-boost with DNA, did not reduce HIV-1 acquisition rates or set-point viral loads in clinical trials [24–27]. Both CD4^+^ and CD8^+^ T-cell responses were detected in the vaccinees, with a predominance of CD8^+^ T-cell responses. Whether the insufficient magnitude, functionality or breadth of the vaccine-induced cellular immune responses participated in the failure of the Ad5 vaccine to provide demonstrable protection against HIV-1 infection remains unclear. In particular, in the ‘STEP’ clinical trial, pre-existing Ad5-specific antibody titers appeared to have negatively impacted the HIV-1-specific CD8^+^ T-cell responder rate after vaccination [26].

The development of non-human primate (NHP) adenovirus-derived vectors may present an alternative to circumvent pre-existing immunity against human adenoviruses. The prevalence of neutralizing antibodies (NAbs) to NHP adenoviruses is markedly lower than that of anti-human adenovirus NAbs [28–31]. In particular, the chimpanzee adenovirus type 7 (AdC7) is thought to circulate minimally in human populations, and appears to be able to circumvent cross-neutralization from pre-existing anti-human adenovirus antibodies [32]. The prevalence of NAbs to AdC7 in human populations has been reported to be low in the United States, Uganda and South Africa, with very weak NAb titers when seropositivity was detected [28,31]. Replication-incompetent chimpanzee adenoviral vectors have been shown to elicit potent CD8^+^ T-cell responses against several transgenes in mice and NHPs [32–43]. We therefore hypothesize that low-seroprevalent, replication-incompetent chimpanzee adenoviral vectors could ultimately induce enhanced protective responses against HIV replication in humans, relative to vectors based on human serotypes.

We have previously reported that the F4/AS01 candidate vaccine, consisting of the F4 recombinant fusion protein containing four HIV-1 clade B antigens (Gag p24, Pol reverse transcriptase [RT], Nef and Gag p17) and the AS01 Adjuvant System, induced potent polyfunctional CD4^+^ T-cell responses in HIV-seronegative volunteers, as well as in HIV-1-infected patients on antiretroviral therapy (ART) [44–46]. Here, we have investigated the immunogenicity of heterologous prime-boost regimens with F4/AS01 and a recombinant AdC7 vector expressing HIV-1 clade B Gag, RT and Nef (AdC7-GRN), when delivered intramuscularly in rhesus macaques and mice. In particular, we explored the impact of sequential administration of these two candidate vaccines on the magnitude, memory phenotype and functional quality of vaccine-induced peripheral blood CD4^+^ and CD8^+^ T-lymphocyte responses in the NHP model, and subsequently used the mouse model to characterize local cell-mediated immune (CMI) responses in various mucosal and systemic anatomical compartments.

## Results

The immunogenicity of the adjuvanted protein F4/AS01 (‘P’) and/or the recombinant adenoviral vector AdC7-GRN (‘A’), when administered in homologous (PP or AA) or heterologous (AAPP or PPAA) prime-boost regimens, was evaluated in rhesus macaques, by assessing vaccine antigen-specific T-cell and humoral responses in peripheral blood. As a complementary analysis, we used similar homologous and heterologous prime-boost regimens to evaluate the localization of vaccine-induced responses in mucosal and systemic anatomical compartments in the mouse model. Data of the individual animals are presented in S1–S8 Tables.

### Prime-boost regimens with F4/AS01 and AdC7-GRN induce HIV-1-specific polyfunctional CD4^+^ and CD8^+^ T-cell responses in macaques

**Fig 1:**
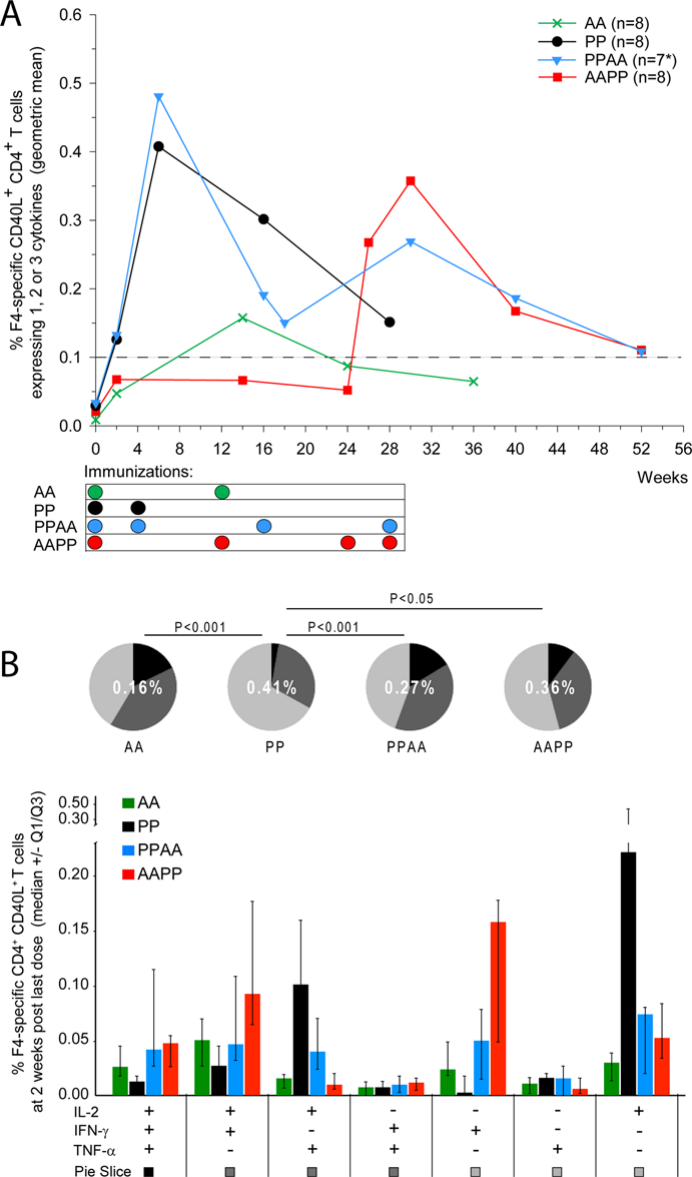
HIV-1-specific CD4+ T-cell responses in NHP. Rhesus macaques were immunized intramuscularly with F4/AS01 (‘P’) and/or AdC7-GRN (‘A’), according to the following regimens: weeks 0 and 4 (PP group), weeks 0 and 12 (AA group), weeks 0, 4, 16 and 28 (PPAA group) and weeks 0, 12, 24 and 28 (AAPP group). **A.** PBMCs were stimulated *in vitro* overnight with a pool of peptides covering the F4 antigen sequence, and production of IL-2, IFN- and TNF- was measured by ICS. Frequency of F4-specific CD4^+^ T cells was expressed as the percentage of CD40L^+^ CD3^+^ CD4^+^ T cells expressing IFN-γ and/or TNF-α and/or IL2 of all CD3^+^ CD4^+^ T cells. Data are represented as group geometric mean frequencies. *: PPAA group: N = 8 at Weeks 0, 2 and 6, and N = 7 at all time-points thereafter. The dashed line indicates the assay cut-off value for CD40L^+^ CD4^+^ T cells (i.e., 0.1%). **B.** The cytokine co-expression profile of F4-specific CD4^+^ T cells at 2 weeks post last immunization is presented for each prime-boost regimen. Data are reported as median frequencies of responding CD40L^+^ CD4^+^ T cells expressing any combination of IL-2, IFN- and TNF-, with first and third quartiles measured. Pie charts represent the group mean proportions of responding CD40L^+^ CD4^+^ T cells expressing (after *in vitro* stimulation) one, two or three cytokines (represented in light grey, dark grey and black, respectively) among IL-2, IFN- and TNF-. These group mean proportions were compared using Fisher’s exact test and a significance level of p<0.05. The number in the center of each pie represents the geometric mean of the total percentage of cytokine-expressing CD40L^+^ CD4^+^ T cells at 2 weeks post last immunization.

HIV-1-specific CD4^+^ and CD8^+^ T-cell responses were detected by flow cytometry. We first evaluated the immunogenicity of homologous prime-boost regimens. Two immunizations with F4/AS01 (at Weeks 0 and 4) elicited HIV-1-specific CD4^+^ T-cell responses in all vaccinated animals in the PP and PPAA groups (geometric means [95% confidence interval, CI] of 0.41% [0.24–0.69] and 0.48% [0.24–0.98], respectively at Week 6), while two AdC7-GRN immunizations (at Weeks 0 and 12) induced low or barely detectable CD4^+^ T-cell responses in the AA and AAPP groups (geometric means [95% CI] of 0.16% [0.11–0.23] and 0.07% [0.05–0.09], respectively, at Week 14; Fig 1A). For both vaccine candidates, CD4^+^ T-cell responses following homologous prime-boost regimens peaked two weeks after the second immunization at Weeks 6 or 14, with HIV-1-specific CD4^+^ T-cell responses that were significantly higher for F4/AS01 than for AdC7-GRN (p<0.0001; Satterthwaite t-test). In contrast to the AdC7-GRN-induced responses, F4/AS01-induced CD4^+^ T-cell responses persisted at 6 months after the second immunization (Weeks 36 and 28 for the AA and PP groups, respectively).

To evaluate the impact of heterologous prime-boost regimens, we compared the magnitudes of HIV-1-specific CD4^+^ T-cell responses before and after the heterologous booster immunizations in animals primed with two homologous vaccinations. The response after the two heterologous booster immunizations with F4/AS01 in AdC7-GRN-primed animals was not significantly different from that after two F4/AS01 immunizations in naïve animals (AAPP group at Week 30 *vs* PP/PPAA groups at Week 6; p>0.05; Fig 1A). In F4/AS01-primed animals of the PPAA group, the priming-induced response initially declined from its peak in Week 6, but two heterologous booster immunizations with AdC7-GRN given at Weeks 16 and 28 (i.e., 3 and 6 months after the second priming immunization with F4/AS01) induced a slight recall of the response.

The cytokine (IL-2/IFN-γ/TNF-α) expression profile of circulating HIV-1-specific CD40L^+^ CD4^+^ T cells was characterized and compared between the four groups at 2 weeks post last immunization (Weeks 6, 14 or 30; Fig 1B). Two weeks after the second priming immunization (Weeks 6 or 14), the proportion of HIV-1-specific polyfunctional (double- or triple-cytokine-expressing) CD40L^+^ CD4^+^ T cells was significantly higher in AdC7-GRN-primed animals than in F4/AS01-primed animals (AA *vs* PP; p<0.0005; Fisher’s exact test), although the total HIV-1-specific cytokine-expressing CD4^+^ T-cell response in the AA group was low (geometric mean [95% CI] of 0.16% [0.11–0.23] at Week 14). In the PP group, the majority of F4/AS01-induced HIV-1-specific CD4^+^ T cells produced only IL-2, and smaller proportions of cells produced IL-2 in combination with TNF-α. Priming with AdC7-GRN had an influence on the cytokine expression profile. Specifically, F4/AS01 administration induced predominantly IL-2^+^ CD40L^+^ CD4^+^ T cells (single-cytokine-expressing or IL-2^+^ TNF-α^+^ cells) when given to naïve animals in the PP group, but predominantly IFN-γ^+^ CD40L^+^ CD4^+^ T cells (single-cytokine-expressing or IL-2^+^ IFN-γ^+^) when given to AdC7-GRN-primed animals in the AAPP group. Moreover, the proportion of double- or triple-cytokine-expressing HIV-1-specific CD40L^+^ CD4^+^ T cells was significantly higher after each of the two heterologous (AAPP or PPAA) prime-boost regimens than after F4/AS01 priming only (p = 0.0461 [AAPP *vs* PP] and p<0.0001 [PPAA *vs* PP]; Fisher’s exact test).

**Fig 2:**
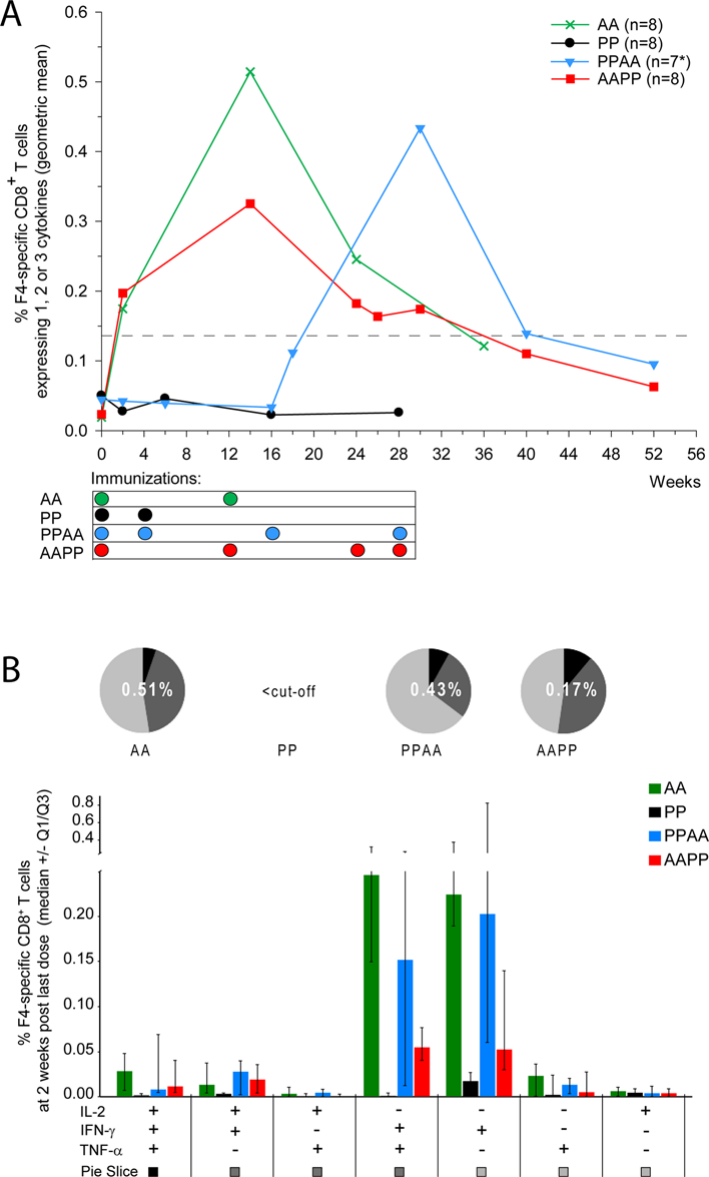
HIV-1-specific CD8+ T-cell responses in NHP. Rhesus macaques were immunized intramuscularly with F4/AS01 (‘P’) and/or AdC7-GRN (‘A’), according to the following regimens: weeks 0 and 4 (PP group), weeks 0 and 12 (AA group), weeks 0, 4, 16 and 28 (PPAA group) and weeks 0, 12, 24 and 28 (AAPP group). **A.** PBMCs were stimulated *in vitro* overnight with a pool of peptides covering the F4 antigen sequence at several time-points, and production of IL-2, IFN- and TNF- was measured by ICS. Frequencies of F4-specific CD8^+^ T cells were expressed as percentages of CD3^+^ CD8^+^ T cells expressing IFN-γ and/or TNF-α and/or IL2 over total CD3^+^ CD8^+^ T cells. Data are represented as group geometric mean frequencies. *: PPAA group: N = 8 at Weeks 0, 2 and 6, and N = 7 at all time-points thereafter. The dashed line indicates the assay cut-off value for CD8^+^ T cells (i.e., 0.14%). **B.** The cytokine co-expression profile of F4-specific CD8^+^ T cells at 2 weeks post last immunization is represented for each prime-boost regimen. Data are reported as median frequencies of responding CD8^+^ T cells expressing any combination of IL-2, IFN- and TNF-, with first and third quartiles measured. Pie charts show the group mean proportions of responding CD8^+^ T cells expressing (after *in vitro* stimulation) one, two or three cytokines (represented in light grey, dark grey and black, respectively) among IL-2, IFN- and TNF-. These group mean proportions were compared using Fisher’s exact test and a significance level of p<0.05. The number in the center of each pie represents the geometric mean of the total percentage of cytokine-expressing CD8^+^ T cells at 2 weeks post last immunization.

No circulating HIV-1-specific CD8^+^ T cells were detected in animals immunized with F4/AS01 only, at any time-point evaluated (Fig 2A). In contrast, the first AdC7-GRN immunization given to naïve animals induced HIV-1-specific CD8^+^ T-cell responses, which increased 2 weeks after the second AdC7-GRN immunization (Week 14) to peak geometric means [95% CI] of 0.51% [0.26–1.04] in the AA group and 0.33% [0.15–0.69] in the AAPP group. Similarly, two AdC7-GRN immunizations given to F4/AS01-primed animals induced strong CD8^+^ T-cell responses, but these were not significantly different from the responses after AdC7-GRN administration in naive animals (PPAA group at Week 30 *vs* AAPP/AA groups at Week 14; p>0.05; Satterthwaite t-test). Very low frequencies of circulating CD8^+^ T-cell responses were still detectable at 6 months post last vaccination (geometric means [95% CI] of 0.12% [0.06–0.23], 0.06% [0.04–0.11] and 0.10% [0.03–0.32] for the AA, AAPP and PPAA groups at Weeks 36, 52 and 52, respectively).

Two weeks after the last dose, the cytokine expression profiles of HIV-1-specific CD8^+^ T cells in the AA, PPAA and AAPP groups were not significantly different from each other (Fig 2B; p>0.05; Fisher’s exact test). The majority of specific CD8^+^ T cells expressed IFN-γ, either alone or in combination with TNF-α.

### HIV-1 antigens targeted by the vaccine-elicited T cells in macaques

**Fig 3:**
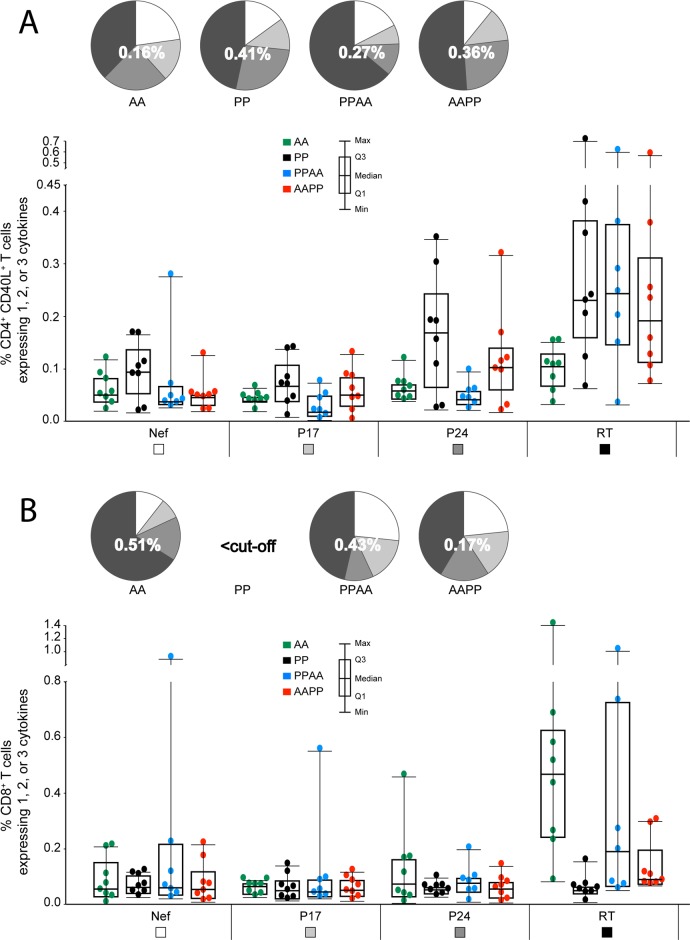
HIV-1 antigens targeted by CD4+/CD8+ T-cell responses in NHP (2 weeks post last immunization). Magnitudes of p24-, p17-, RT- and Nef-specific CD40L^+^ CD4^+^ T-cell responses (panel A) and CD8^+^ T-cell responses (panel B) expressing IL-2, IFN- and/or TNF- were assessed at 2 weeks post last immunization using ICS. PBMCs were stimulated *in vitro* overnight with a pool of peptides covering the p24, p17, RT or Nef sequences. Individual data and box plots, showing the medians, upper and lower interquartiles and minimum and maximum values, are represented for each group. Pie charts show the mean proportions of CD40L^+^ CD4^+^ or CD8^+^ T cells specific to Nef, p17, p24 and RT (represented in light grey, medium grey, dark grey and black, respectively). The number in the center of each pie represents the geometric mean of the total percentage of cytokine-expressing CD40L^+^ CD4^+^ or CD8^+^ T cells at 2 weeks post last immunization.

Proportions of cytokine (IL-2/IFN-/TNF-)-expressing p24-, p17-, RT- and Nef-specific CD40L^+^ CD4^+^ and CD8^+^ T cells among all cytokine-expressing CD40L^+^ CD4^+^ and CD8^+^ T cells were assessed by intracellular cytokine staining (ICS) at 2 weeks post last immunization (Fig 3).

CD4^+^ T-cell responses were mainly targeted against RT, then p24, then p17 or Nef, irrespective of the prime-boost regimen followed, although this pattern appeared to be more prominent in the heterologous prime-boost groups. CD8^+^ T-cell responses were also predominantly targeted against RT, with low and comparable proportions directed to each of the three other HIV-1 antigens.

### Phenotypical and functional characterization of the induced HIV-1 specific memory T cells in macaques

Next, we analysed the memory phenotypes of peripheral HIV-1-specific memory CD4^+^ and CD8^+^ T-cell responses 6 months after the last immunization, using the memory marker CD95 and the costimulatory molecule CD28 to classify vaccine-elicited T cells as effector memory (T_EM_; CD28^-^ CD95^+^) or central memory (T_CM_; CD28^+^ CD95^+^) subsets [47,48].

**Fig 4:**
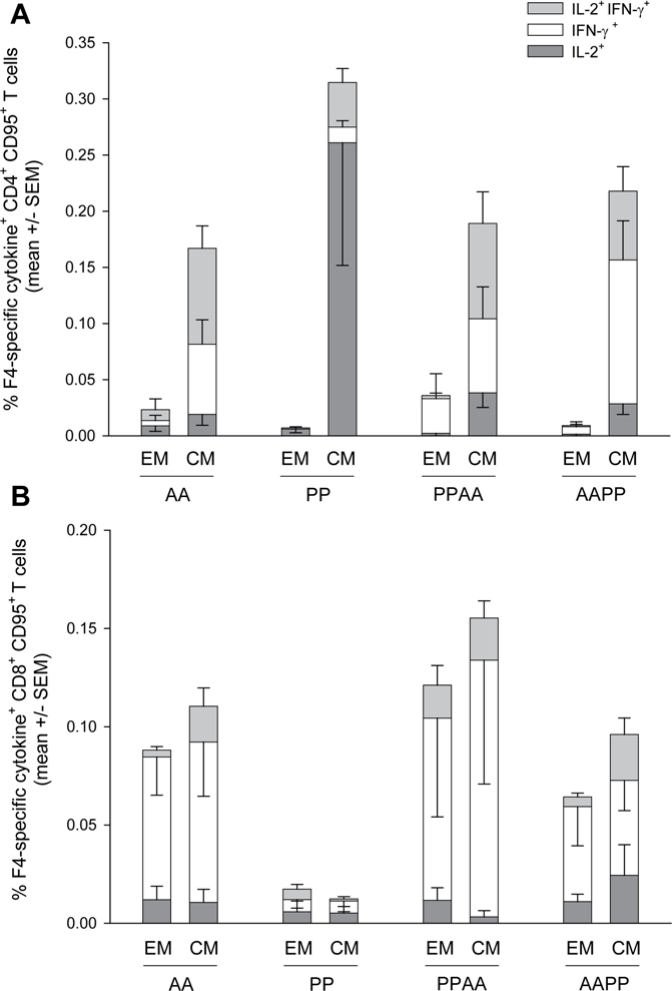
Phenotype of memory HIV-1-specific T cells in NHP at 6 months post last immunization. Frequencies and cytokine expression profile of F4-specific central memory (‘CM’; CD28^+^ CD95^+^) and effector memory (‘EM’; CD28^-^CD95^+^) CD4^+^ and CD8^+^ T cells were determined at 6 months post last immunization by ICS after overnight *in vitro* stimulation of PBMCs with a pool of peptides covering the F4 sequence. Data are represented as group mean percentages +/- standard error of the mean (SEM).

While the memory CD4^+^ T-cell responses induced by each of the prime-boost regimens comprised mostly T_CM_ cells, the memory CD8^+^ T-cell responses suggested a balance between T_CM_ and T_EM_ phenotypes (Fig 4), although the geometric mean frequencies of the HIV-1 specific CD8^+^ T cells were very low (range 0.03–0.12%). The observed T-cell cytokine expression profiles were comparable to those seen at 2 weeks post last vaccination, with predominantly IL-2-expressing CD4^+^ T-cell responses in the PP group, and more balanced expression profiles for the CD4^+^ T-cell responses in the other groups and for the CD8^+^ T-cell responses in the AA, PPAA and AAPP groups (Figs 1B and 2B).

### Prime-boost regimens induce F4-specific antibody responses in macaques

**Fig 5:**
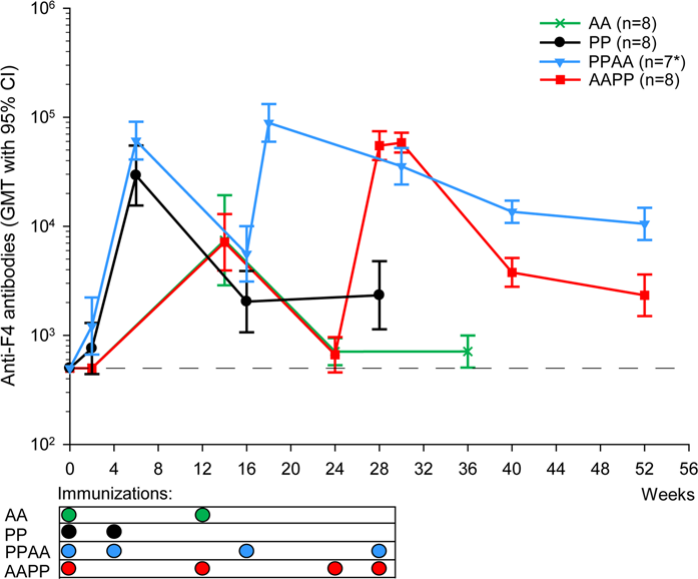
Humoral responses against the F4 antigen in NHP. Anti-F4 binding antibody titers were measured by ELISA and presented as geometric mean titers (GMT), with 95% confidence interval (CI). *: PPAA group: N = 8 at Weeks 0, 2 and 6, and N = 7 at all time-points thereafter. Sera from non-responding animals were assigned a value of 500, as indicated by the dotted line.

F4-specific binding antibody responses were assessed by ELISA (Fig 5). While the responses were absent or low in all groups after the first immunization at Week 2 (geometric mean titres [GMTs] ≤1217), they increased sharply after either of the second homologous immunizations, with GMTs that were significantly higher in F4/AS01 recipients than in AdC7-GRN recipients (PP/PPAA groups at Week 6 *vs* AAPP/AA groups at Week 14; p<0.0001; Satterthwaite t-test).

GMTs were also compared after the first heterologous immunization. In F4/AS01-primed animals, one AdC7-GRN immunization significantly boosted the GMT (PPAA group at Week 18 *vs* PP/PPAA groups at Week 6; p = 0.020). Similarly, in AdC7-GRN-primed animals, one dose of F4/AS01 significantly boosted the AdC7-GRN-induced response, but did not induce a higher response relative to that observed after F4/AS01 priming in naïve animals (AAPP group at Week 26 *vs* PP/PPAA groups at Week 6; p>0.05).

In all groups, GMTs declined about 10 to 25-fold from their peaks over time but persisted until at least six months after the last immunization, with the highest response (GMT = 10515) observed in F4/AS01-primed animals boosted with AdC7-GRN (PPAA group) at Week 52.

### High-frequency responses of HIV-1-specific T cells in systemic and mucosal tissues of mice

Last, we sought to determine the anatomic localization of vaccine-induced T-cell responses at one week post last immunization, using the mouse model. CB6F1 mice were immunized following homologous (PP or AA) or heterologous (AAPP or PPAA) prime-boost regimens, or received saline (control). We evaluated the magnitudes of F4-specific cytokine (IL-2/IFN-γ/TNF-α)-producing CD4^+^ and CD8^+^ T-cell responses in liver and spleen, as well as in mucosa of the genital tract and small intestine. Due to the small number of data points, no comparative statistical analyses between groups were performed.

**Fig 6:**
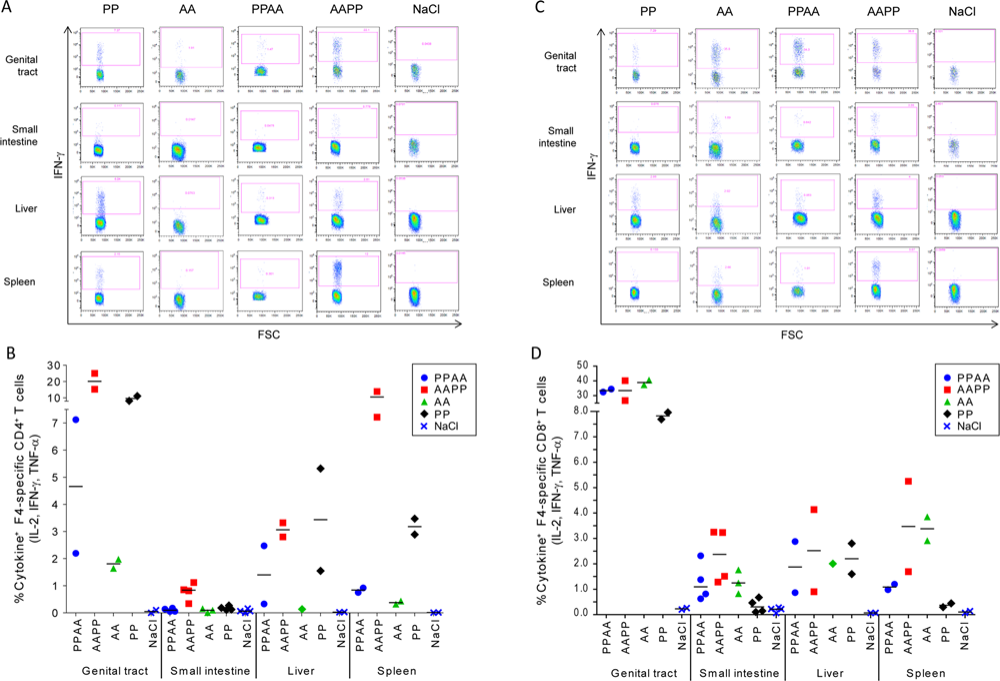
Local T-cell responses in CB6F1 mice. CB6F1 mice (N = 40/group) were immunized intramuscularly with two administrations (2 weeks apart) of F4/AS01 (‘P’) and/or AdC7-GRN (‘A’) following the PP, AA, PPAA and AAPP prime-boost regimens, or received saline. Seven days after the last immunization, the presence of F4-specific CD4^+^ and CD8^+^ T cells in the liver, spleen or mucosa from genital tract and small intestine was assessed by stimulating cells *in vitro* 4 hours with a pool of peptides covering the F4 sequences, and analysis using ICS. A/C panels: Representative dot plot graphs of CD4^+^ (A panel) and CD8^+^ (C panel) T-cell responses. Local CD4^+^ and CD8^+^ T cells were analyzed with respect to the production of IFN-γ (shown from a representative sample), IL-2 and TNF-α (not shown). The numbers in the quadrant gates of the plots represent the frequency of IFN-γ producing cells. FSC, forward scatter. B/D panels: Frequencies and cytokine expression profile of F4-specific CD4^+^ (B panel) and CD8^+^ (D panel) T cells expressing at least one cytokine among IL-2, IFN- and TNF- localized in the different anatomical compartments are represented as individual data and group means. Each symbol corresponds to a pool containing samples of 20 animals (genital tract), 5 animals (small intestine) or 3 animals (liver), or to a sample from 1 animal (spleen).

While no F4/AS01-induced CD8^+^ T-cell responses were observed in our experiments in the NHP model (as described above), such responses were observed in CB6F1 mice. After vaccination, HIV-1 specific cytokine-expressing CD4^+^ and/or CD8^+^ T cells were detected in each compartment assessed, with the highest frequencies found in the genital tract mucosa (Fig 6). No CD4^+^ or CD8^+^ T-cell responses were detected in the control group.

CD4^+^ T-cell responses were observed in the spleen, liver and genital tract of animals immunized with the PP, AAPP and PPAA regimens, but only the AAPP regimen elicited specific CD4^+^ T cells in the mucosa of the small intestine (Fig 6A and 6B). HIV-1-specific CD8^+^ T-cell responses were detected in each compartment and with each vaccine regimen (Fig 6C and 6D). In each compartment, mean frequencies of CD8^+^ T-cell responses were comparable between the vaccine regimens comprising the adenoviral vector (ie., AA, AAPP or PPAA), and the mean responses induced by these regimens were generally higher than those induced by the PP regimen.

## Discussion

HIV-1-specific polyfunctional CD4^+^ and CD8^+^ T-cell responses are thought to contribute to the control of viral replication in SIV-infected NHP and HIV-infected humans [1–5]. While CD8^+^ T cells have been associated with some control of HIV-1 and SIV replication [4,5,49], CD4^+^ T cells are implicated in maintaining functional HIV-specific responses of memory CD8^+^ T cells and antibodies [6–8,50]. Developing prophylactic HIV-1 vaccine regimens that are able to elicit balanced T-cell responses consisting of both CD4^+^ and CD8^+^ T cells might therefore offer a valuable contribution to the antibody-based vaccine strategies. The candidate vaccine F4/AS01 has been shown to induce robust polyfunctional CD4^+^ T-cell responses in HIV-seronegative volunteers and HIV-1-infected patients on ART, and lower levels of these responses in ART-naïve HIV-1-infected subjects [44–46]. The present study in NHP and mice aimed to assess the immunogenicity and functionality of CMI responses induced by heterologous prime-boost regimens using the adjuvanted protein vaccine F4/AS01 and the chimpanzee recombinant adenoviral vector AdC7-GRN. This simian vector was used based on its very low prevalence in humans, and the capacity of adenoviral vectors in general to elicit potent CD8^+^ T-cell responses. The data generated in the NHP model could facilitate extrapolation to the clinical setting, while the murine data allow for an exploratory characterization of the anatomical localization of the responding T cells induced by the different immunization regimens.

Our results show that: (i) In NHP, both heterologous prime-boost regimens induced polyfunctional HIV-1-specific CD4^+^ and CD8^+^ T-cell responses; (ii) AdC7-GRN priming or boosting increased the polyfunctionality, but not the frequency of HIV-1-specific CD4^+^ T-cell responses elicited by F4/AS01. HIV-1-specific CD8^+^ T-cell responses were mainly driven by AdC7-GRN, and no influence of priming or boosting with F4/AS01 was seen on the cytokine expression profiles of responding CD8^+^ T cells; (iii) Each of the four HIV-1 antigens of F4 was recognized by the vaccine-induced CD4^+^ and CD8^+^ T cells; (iv) Approximately half of the vaccine antigen-specific CD8^+^ T-cell responses exhibited a T_EM_ phenotype; and (v) In mice, HIV-1-specific T-cell responses were detected in mucosal and systemic anatomical compartments.

HIV-1-specific CD4^+^ T-cell responses were primarily driven by F4/AS01. This is in accordance with clinical data, showing that the vaccine induces predominantly CD4^+^ T-cell responses in humans [44–46], and with the fact that AS01 is a potent enhancer of T-cell responses, as demonstrated in various clinical studies with a range of antigens (reviewed in [51,52]). In contrast to vaccine antigen-specific CD4^+^ T-cell responses, specific CD8^+^ T-cell responses were only detected in recipients of AdC7-GRN, and were comparable between F4/AS01-primed and naïve macaques. The CD8^+^ T-cell response against HIV-1 antigens supports observations made with other replication-incompetent simian adenoviral vectors expressing HIV-1 or SIV antigens in preclinical models [32–37,39,53]. Taken together, only the heterologous prime-boost regimens allowed for a balanced response of both CD4^+^ and CD8^+^ T cells, with the two candidate vaccines acting complementarily rather than synergistically. After one AdC7-GRN administration, the magnitude of the HIV-specific CD8^+^ T-cell response was comparable to that observed after one administration of a SIV antigen-expressing AdC7 or AdC6 vector in macaques in a recent study [54]. However, after a second AdC7-GRN immunization, the CD8^+^ T-cell response measured in the current study appeared to be less potent than the response observed after a heterologous second immunization with AdC6 or AdC7 vectors in ref. [54]. Potentially, the use of alternating AdC vectors in ref. [54] circumvented the induction of low-level adenoviral vector-specific nAb responses, which may have been present after AdC7-GRN immunization in our study. Boosting with poxvirus-based vectors (MVA or NYVAC) after AdC vector priming has also been shown to efficiently increase the frequency of insert-specific CD8^+^ T cells [22,39,40,42,55]. In NHP or in mice, AdC and Ad5 vectors generally appeared to induce comparable magnitudes of HIV-specific CD8^+^ T cells [32,56]. However, the responses differed in terms of memory phenotypes and cytokine expression profiles, with a higher level of functional exhaustion and a relatively high proportion of single cytokine-expressing T cells after Ad5 vaccination [39,57]. Adenoviral vectors, particularly after a single administration, may generally be more immunogenic in NHP or mice as compared to MVA vectors or DNA vaccines (which require multiple administrations to induce a potent CD8^+^ T-cell response [58–61]). However, when used in prime-boost, a DNA prime-NYVAC boost regimen has been shown to induce a robust HIV-specific CD8^+^ T-cell response in macaques [62], with a magnitude comparable to that observed with the AdC7 vector in the present study, though the magnitude of the induced CD4^+^ T-cell response tended to be lower than the one obtained with an adjuvanted protein in the current study. Lastly, in our study, the sequence of vaccine administration did not have an impact on the magnitude of the observed responses persisting at 6 months post last vaccination.

Both candidate vaccines, when administered individually, were able to induce polyfunctional T-cell responses. Moreover, when administered in a heterologous prime-boost schedule, priming or boosting with AdC7-GRN altered the F4/AS01-induced immune profile (which was dominated by single cytokine-expressing IL-2^+^ CD4^+^ T cells), by increasing the frequencies of polyfunctional CD4^+^ T cells expressing IL-2 in combination with IFN-γ and/or TNF-α. This is encouraging since HIV patients with a ‘controller’ status were shown to have higher proportions of Gag-specific CD4^+^ T cells producing 3 cytokines than other HIV patient categories [63], and polyfunctional HIV-specific CD4^+^ and CD8^+^ T cells have been associated with long-term non-progressing disease and low viral load in humans [9–13,63]. The predominance of IL-2-secreting cells among F4/AS01-induced CD4^+^ T-cell responses, as found in the present study, is in line with previous clinical results with F4/AS01 [44,45]. While IL-2 production predominance is a common feature of adjuvanted protein vaccines [64], it has also been linked to favorable clinical outcomes in HIV-infected patients [10,65,66]. IL-2 is associated with memory T cells, as well as T-cell proliferation and differentiation [10,67].

Although each of the HIV-1 antigens was recognized, the specific cytokine-expressing CD4^+^ and CD8^+^ T-cell responses were mainly directed against RT and p24. This distribution of antigenic specificities as well as the cytokine expression profiles of the F4/AS01-induced CD4^+^ T-cell responses, were in agreement with the features of the CD4^+^ T-cell responses seen in human F4/AS01 recipients [44]. Although the predominance of RT and p24-specific responses could be a function of the respective lengths of the antigen sequences in F4, and/or associated number of epitopes contained therein, this result is still promising since the same CD4^+^ T-cell specificity distribution was seen among responses in HIV-infected patients with spontaneous viral control [68]. Illustrating the importance of Gag-specific responses, the frequency of polyfunctional Gag-specific CD8^+^ T cells was found to be highly associated with the ability of CD8^+^ T cells to control SIV and HIV-1 viremia [10,23,49,69,70].

Six months after the last immunization, the induced memory CD4^+^ T cells were mainly T_CM_-biased, while approximately half of the induced memory CD8^+^ T cells maintained an activated (T_EM_-biased) phenotype. Similar results were found with Gag-expressing chimpanzee adenoviral vector prime-boost regimens in NHP, which induced a balance between CD8^+^ T_EM_ and T_CM_ cells [32]. CD8^+^ T_EM_-cell responses have been shown to be associated with the control of pathogenic SIV challenge, with some evidence of viral clearance [4,5,71]. Therefore, a vaccine that elicits persistent specific T_EM_-biased responses, particularly at mucosal sites, may be able to contribute to initial viral control. However, T_CM_ cells, which have limited immediate effector functions, are known to replenish the T_EM_ cell pool in a dynamic turnover, and may therefore also contribute to protection. Indeed, in elite controllers who had maintained only minimal CD8^+^ T-cell responses, highly functional, broad-spectrum T_CM_ cells were detected, which were shown to suppress HIV *in vitro* [72].

In mice, each of the prime-boost regimens generated CD4^+^ and CD8^+^ T-cell responses across multiple systemic and mucosal anatomical compartments, in line with studies using different adenoviral vectors in mice or NHP [18,19,73,74]. Responses were higher in genital tract mucosa as compared to intestinal mucosa or systemic organs (liver and spleen), as also observed with other adenoviral vectors [18,19,73]. Maintenance of robust mucosal CD8^+^ T-cell responses is important for viral control in NHP [75–78], as well as in humans for whom it has been associated with a favourable clinical outcome [79–82]. Furthermore, due to haematogenous viral dissemination, maintenance of functional CD8^+^ T-cell responses in local and systemic compartments is also considered to be important for infection control in NHP [5]. Whether or not our findings in mice would translate to NHP or humans remains to be determined. However, the distribution of mucosal cellular responses among anatomical compartments after IM immunization with Ad vector-based SIV vaccines was previously shown to be comparable between these two models [18,19,73].

HIV-specific binding antibody responses were detected in all groups and seemed to be driven by each of the two vaccines. This is in line with the potent HIV-specific binding antibody responses seen in several clinical studies, either with F4/AS01 [44] or with HIV antigen-expressing adenovectors [83,84]. Recent findings indicate that HIV-1 envelope-specific non-neutralizing IgG antibodies may be important for protection against HIV or simian-human immunodeficiency virus acquisition [22,23,85,86], suggesting that an HIV-1 prophylactic vaccine should also contain envelope-based antigens. Therefore, the development of alternative prime-boost approaches with chimpanzee adenoviral vectors and adjuvanted proteins, in combination with any strategies aiming at inducing HIV-1 envelope-specific antibodies, might merit further exploration.

## Conclusion

Heterologous prime-boost regimens with F4/AS01 and AdC7-GRN candidate vaccines induced balanced polyfunctional CD4^+^ and CD8^+^ T-cell responses and HIV-specific IgG antibodies in peripheral blood of NHP, and local T-cell responses in various mucosal and systemic compartments in mice. This suggests that prime-boost regimens combining adjuvanted protein and low-seroprevalent simian adenoviral vectors may represent attractive vaccination strategies for prophylactic HIV-1 vaccines.

## Materials and Methods

### Vaccine candidates

The F4/AS01 HIV-1 vaccine candidate has been described previously [44,45,68]. Briefly, the F4 antigen is a recombinant fusion protein expressed in *Escherichia coli* containing the HIV-1 subtype B antigens p24 (BH10), RT (HXB2), Nef (Bru-Lai) and p17 (BH10). F4 was formulated in the liposome-based AS01_B_ Adjuvant System (elsewhere in this article referred to as AS01) containing 50 μg MPL and 50 μg QS21 (*Quillaja saponaria* fraction 21; Antigenics Inc., a wholly owned subsidiary of Agenus Inc.) per human dose (500 μL).

We have generated an E1- and E3-deleted AdC7 adenovirus vector based on the ATCC sequence #VR-593. An insert was introduced in the AdC7 molecular clone under the control of a CMV IE promoter to express a fusion protein consisting of the same HIV-1 subtype B antigens as in the F4 protein, in the order Gag-RT-Nef (GRN). The recombinant replication-incompetent AdC7-GRN viruses were produced by transfection of HEK 293 packaging cells and purified on a CsCl gradient.

### Animals and immunizations

The NHP study was carried out in strict accordance with the European regulations regarding the protection of animals used for experimental purposes, in particular Council directive N° 86/609/EEC. Before study initiation, the protocol for the NHP study was submitted for ethical review and approved by the institutional Ethical Committee of the Centre International de Toxicologie (CIT; Evreux, France; Association for Assessment and Accreditation of Laboratory Animal Care (AAALAC)-accredited). This review has been documented in the Ethical Committee database (reference number 00863).

Sixteen female and 16 male, Chinese-origin, purpose-bred rhesus macaques (*Macaca mulatta*) of approximately 30 months old were housed at CIT in a dedicated primate unit (temperature of 24°C± 3°C; humidity of 50± 20%; light/dark cycle of 12 h/12 h). Animals were housed in custom-build group-housing cages (L:1.55 m × D: 1.55m × H: 2.5m; Volume: 6 m^3^) with grid floors (up to 6 animals/cage). All macaques had free access to tap water and received a daily ration of 180 g of OWM (E) SQC SHORT expanded diet (Dietex France). In addition, fruits and/or vegetables and plastic toys were given daily as enrichment. Mean body weights were 3 kg (range: 2.3 to 4.2 kg) for males and 2.7 kg (range: 2.2 to 3.1 kg) for females.

Macaques were allocated to four groups of 4 males and 4 females each, based on their levels of non-specific T-cell responses observed in the ICS assay at pre-vaccination, to ensure a balanced distribution among groups. They received two intramuscular (IM) injections of AdC7-GRN (‘A’) and/or F4/AS01 (‘P’) following homologous (PP or AA) or heterologous (PPAA or AAPP) prime-boost regimens, as indicated in Table 1. Injections were administered intramuscularly and contained 1 × 10^11^ viral particles (vp) of AdC7-GRN and/or 90 μg of F4 protein formulated in 500 μL AS01. Monkeys were bled without anaesthesia before and at several time-points post immunization (Table 1). A moribund female (of the PPAA group) was prematurely sacrificed by exsanguination at day 66, after sedation with ketamine hydrochloride and anaesthesia with thiopental. The animal showed dehydratation, hypoactivity and marked liquid feces from day 65, and thin appearance, hypothermia, ventral recumbency, prostration, enophtalmus and half-closed eyes on the day of sacrifice. Coprology showed the presence of a few *Trichuris Trichiura* eggs, but the observed quantity did not explain the clinical symptoms. Subsequent macroscopic and microscopic *post-mortem* examinations indicated that the animal’s poor health condition was not causally related to the immunizations, but did not conclusively reveal the cause of death. All other monkeys were euthanized humanely by an overdose of anesthetic after completion of the experiments.

**Table 1:** Prime-boost regimens and immunization schedules for the NHP study.

Group	N	Prime-boost regimen	Immunization time-points (Week)	Blood sampling time-points (Week)
AA	4M/4F	2 × AdC7-GRN	0	0, 2
			12	14, 24, 36
PP	4M/4F	2 × F4/AS01	0	0, 2
			4	6, 16, 28
AAPP	4M/4F	2 × AdC7-GRN + 2 × F4/AS01	0	0, 2
			12	14
			24	24, 26
			28	30, 40, 52
PPAA	4M/3F^a^	2 × F4/AS01 + 2 × AdC7-GRN	0	0, 2
			4	6, 16
			16	18
			28	30, 40, 52

All vaccines were administered intramuscularly.

^a^A moribund female was euthanized at day 66: N = 8 for assays done at Weeks 0, 2 and 6, and N = 7 for all time-points thereafter.

The mouse study was carried out in strict accordance with the European regulations (in particular Council directive N° 86/609/EEC) regarding the protection of animals used for experimental purposes, in GSK’s animal facilities in Rixensart, Belgium (AAALAC-accredited). The GSK institutional ethical committee reviewed the protocol (approval number 07/142/01/A).

Female CB6F1 mice (a hybrid between C57Bl/6 and BALB/C mice) were aged 6–8 weeks at study initiation. The animals had free access to water and a maintenance diet. Consistent with the AAALAC global enrichment program, their environment included nesting material (Envirodry), and social housing was applied.

Mice were randomly allocated to 4 groups (N = 40/group) to receive two or four intramuscular injections (in the gastrocnemian muscle) each containing 50 μL (9 μg) F4 protein formulated in AS01 (‘P’) and/or 1 × 10^8^ vp AdC7-GRN (‘A’). Injections were given according to the following immunization schedules: PP or AA at Days 0 and 14, and AAPP or PPAA at Days 0, 14, 28 and 42. A fifth group (N = 40) received saline only (controls). Seven days after the last immunization, mice were euthanized with a barbituric acid derivative, in order to collect the organs (livers, spleens, small intestines and genital tracts).

### Cell-mediated responses in macaques

Peripheral blood mononuclear cells (PBMCs) were isolated from blood samples using Lymphoprep (Axis-Shield) density gradient centrifugation (700 × *g* for 20 min at 20°C), frozen in 90% fetal calf serum (FCS; Gibco) and 10% dimethyl sulfoxide (DMSO), and cryopreserved. F4-specific CD40L^+^ CD4^+^ and CD8^+^ T cells expressing IFN-γ, IL-2 and/or TNF-α were detected using intracellular cytokine staining (ICS) and flow cytometry. PBMCs were thawed in complete RPMI 1640 medium (Sigma) supplemented with 5% FCS, and plated on 96-well round bottom plates (1 × 10^6^ cells/well). Cells were then stimulated *in vitro* overnight (37°C, 5% CO_2_) in 200 μL medium alone (negative control), with either staphylococcal enterotoxin B (1 μg/mL; Sigma; positive control), peptide pools (15-mers overlapping by 11 amino acids; 1 μg/mL/peptide) covering the full F4 sequence, or with the individual sequences of p24, RT, Nef or p17 antigens at 1 μg/mL/peptide. All cultures contained 1 μg/mL of co-stimulatory molecules anti-CD28 and anti-CD49d (BD Biosciences). After 2 hours of stimulation, Brefeldin A (BD Pharmingen) was added overnight. Cells were then washed in Flow Buffer (phosphate-buffered saline (PBS) 1X, 1% FCS, 0.02% azide) and stained for 40 min at 4°C with monoclonal antibodies (mAbs) specific for surface molecules: anti-CD3-Alexa700 (clone SP34-2), anti-CD4-PerCP (clone L200) and anti-CD8-Pacific Blue (clone RPA-T8) antibodies (all BD Biosciences, USA). Cells were then washed in Flow buffer, permeabilized with Cytofix/Cytoperm solution (BD Biosciences) at 4°C for 20 min, washed with Perm/Wash buffer (BD Biosciences) and then stained for 2 hours at 4°C with mAbs specific for intracellular cytokines and CD40L (i.e., anti-CD154-PE (clone TRAP1), anti-IL2-APC (clone MQ1-17H12), anti-IFN-γ-FITC (clone B27) and anti-TNF-α-PE-Cy7 (clone Mab11) antibodies (all BD Biosciences). Cells were washed again in Perm/Wash buffer and resuspended in CellFIX solution (BD Biosciences). The mAbs used for the memory T-cell phenotype analysis included anti-CD3-Alexa700 (clone SP34-2), anti-CD4-PerCP (clone L200), anti-CD8-APC-Cy7 (clone SK1), anti-CD28-PE (clone CD28.2), anti-CD95-Pacific Blue (clone DX2), IL2-APC (clone MQ1-17H12) and anti-IFN-γ-FITC (clone B27) antibodies (all BD Biosciences).

Stained cells were analyzed by flow cytometry using an LSRII flow cytometer and FACSDIVA (BD Biosciences) or SPICE software (Tree Star, Inc; version 5.2). ICS results were presented as the background-subtracted percentages of CD40L^+^ CD4^+^ and CD8^+^ T cells expressing combinations of IFN-γ, IL-2 and/or TNF-α. F4-specific T-cell responses were considered to be positive if the antigen-specific response was greater than or equal to the cut-off value. Cut-off values were calculated on the basis of the maximum value (rounded to the superior hundredth) among all 95^th^ percentiles of the cytokine-expressing CD40L^+^ CD4^+^ or CD8^+^ T cells following stimulation with F4 peptides at the pre-vaccination time point, and were 0.1% for CD40L^+^ CD4^+^ T cells and 0.14% for CD8^+^ T cells.

### Humoral immune response in macaques

F4-specific binding antibody responses in monkey serum samples were assessed using an enzyme-linked immunosorbent assay (ELISA). Briefly, 96-well plates were coated with the F4 antigen at 0.25 μg/mL in PBS, left overnight at 4°C and then blocked for 1 h at 37°C with saturation buffer (PBS, 0.1% Tween 20, 1% BSA, 4% NCS). Serum samples were serially diluted (twelve dilutions) in saturation buffer and incubated on plates (1.5 h at 37°C). After washing steps (0.1% Tween in PBS), a secondary horseradish peroxidase-labelled mouse anti-human IgG antibody (Stratech) was incubated on plates at a dilution of 1/2000 for 1 h at 37°C. Antibody binding was revealed by addition of the 3,3',5,5'-tetramethylbenzidine substrate (Biorad), and the reaction was stopped by addition of 0.2 M H_2_SO_4_. Optical densities were recorded at 450–620 nm using an Emax microplate reader (Molecular Devices). Mid-point titres were calculated as the reciprocal dilution for which 50% maximal binding was achieved, using SoftMax Pro software v3.1 (Molecular Devices). For GMT calculations, sera from non-responding animals were assigned a value corresponding to half of the lowest dilution used for the serial serum dilutions (i.e., a titre of 500).

### Lymphocyte isolation from mice

Livers, spleens and mucosa of the genital tracts and small intestines from mice were collected at 7 days post the last dose. Small intestine specimens were washed in PBS 1X supplemented with 1% FCS and cut into small pieces, which were then incubated in complete RPMI 1640 medium (Sigma) supplemented with 5% FCS and 2 mM EDTA at 37°C for 3 times 10 min under vigorous shaking. Every 10 min, the medium was renewed to remove epithelial cells. After the third incubation, the specimens were washed twice with fresh medium, and incubated in complete RPMI 1640 supplemented with 0.0625 IU/ml Liberase, 25 mg/mL DNase I (both Roche) and 10% FCS for 30 min at 37°C with vigorous shaking. The digested samples were then filtered through a 100-μm cell strainer, washed with fresh RPMI medium supplemented with 5% FCS. Per group, 4 pooled samples, each containing the cells from 5 animals, were formed. Cells from each sample were resuspended in 30% Percoll (GE Healthcare), layered over a Percoll gradient with 40% and 75% layers and centrifuged at 1800 rpm for 20 min at room temperature. Lymphocytes from intestinal mucosal tissues were collected at the 40%-75% interface and washed twice with RPMI medium supplemented with 5% FCS.

Genital tracts were cut into small pieces and digested by two serial incubations of 30 min at 37°C with complete RPMI 1640 medium supplemented with 10% FCS, 0.0625 IU/mL Liberase and 25 mg/ml DNase I with vigorous shaking. At the end of each incubation period, supernatants were filtered through a 100-μm cell strainer and washed with fresh RPMI 1640 medium supplemented with 5% FCS. Cells from the two incubation periods were pooled, and ultimately, cells from the digestion of 20 genital tracts were pooled (resulting in 2 pooled samples per group). Cells from each sample were then resuspended in 30% Percoll and lymphocytes were isolated through a 40–75% Percoll gradient, as described above.

Before processing, livers were perfused to remove blood. Liver specimens were cut in small pieces and placed in a gentleMACS C tube (Miltenyi Biotech) with complete RPMI medium supplemented with 10% FCS, 0.0625 IU/mL Liberase and 25 mg/mL DNase I. The tissues were dissociated using a gentleMACS dissociator, and at the end of the program, the tube was incubated for 30 min at 37°C with vigorous shaking to complete the digestion. The sample was then filtered through a 100-μm cell strainer and washed with fresh RPMI 1640 medium supplemented with 5% FCS. Cells from 3 livers were pooled (resulting in 2 pooled samples per group), resuspended with 30% Percoll, and lymphocytes were isolated through a 40–75% Percoll gradient, as described above.

Two spleens per group were collected and processed separately. Leukocytes from each spleen biopsy were isolated in complete RPMI 1640 medium using a 7-mL Potter tissue grinder. The homogenized tissue was then filtered over a 100-μm filter (BD Falcon), and the cell suspension was resuspended in 35 mL complete RPMI and centrifuged at 1200 rpm for 10 min. Pellets were resuspended in 50 mL complete RPMI, centrifuged again (1200 rpm, 10 min), resuspended in 5 mL RPMI 1640 supplemented with 5% FCS, and processed for assays.

### Local cell-mediated responses in mice

F4-specific CD4^+^ and CD8^+^ T-cell responses in livers, spleens and mucosa of the vaginal tract and small intestines from mice were assessed at 7 days post last dose. Fresh cells were plated on round bottom 96-well plates (NUNC, Thermo Fisher) at approximately 1 × 10^6^ cells/well. Leukocytes were stimulated *in vitro* for 4 hours (37°C, 5% CO_2_) with peptide pools (15-mers overlapping by 11 amino acids; 1 μg/mL/peptide) covering the full F4 sequence or left unstimulated, in the presence of anti-CD28 and anti-CD49d at 1 μg/mL (BD Biosciences). Brefeldin A was added during the 4 hours (BD Biosciences) and plates were then transferred at 4°C, and left overnight. Next, cells were stained and analyzed using a 5-colour ICS assay. Cells were transferred to V-bottom 96-well plates (NUNC, Thermo Fisher), centrifuged (189 × *g*, 5 min, 4°C) and resuspended in 50 μL Flow Buffer (PBS 1X, 1% FCS, 0.02% azide) containing anti-mouse (m) CD16/32 (clone 2.4G2, 1/50), for 10 min at 4°C. Then, 50 μL Flow Buffer containing anti-mCD4-Pacific Blue (clone RM4-5) and anti-mCD8-PerCp-Cy5.5 (clone 53–6.7) antibodies (final dilution 1/50 each; BD Biosciences) was added for 30 min at 4°C. Cells were pelleted (189 × *g*, 5 min, 4°C), washed with 200 μL Flow Buffer, fixed and permeabilized by adding 200 μL of Cytofix/Cytoperm solution for 20 min at 4 °C (BD Biosciences, USA). Cells were centrifuged (189 × *g* for 5 min at 4°C) and washed with 200 μL Perm/Wash buffer (BD Biosciences, USA). After an additional centrifugation step, cells were stained in 50 μL Perm/Wash buffer with anti-IL2-FITC (clone JES6-5H4, 1/50), anti-IFN-γ-APC (clone XMG1.2, 1/50) and anti-TNF-α-PE (clone MP6-XT22, 1/700) antibodies (BD Biosciences), for 2 h at 4°C. Cells were washed twice with the Perm/Wash buffer harvested in 300 μL BD Stabilizing Fixative solution. Stained cells were analyzed by flow cytometry using a LSRII flow cytometer (BD Biosciences) and FlowJo software (Tree Star, Inc).

### Statistical analysis

SAS version 9.2 was used to perform statistical analysis. The sample size calculation for the NHP study was based on information gathered in previous studies. Considering an alpha risk of 0.05; a standard deviation of 0.3 and 4 groups, it was estimated that with 8 animals/group, a ratio of 3.4-fold between 2 groups in the intensity of T-cell responses could be significantly observed with 80% power. For the analysis of results, means of the log_10_-transformed T-cell frequencies and anti-F4 antibody titers at different time-points were compared between groups using the Satterthwaite t test assuming unequal variances. Proportions of cells expressing one, two or three different cytokines in individual animals were normalized to total 100%, and group mean proportions were compared using Fisher’s exact test. A significance level of p<0.05 was used.

## Supporting Information

S1 Table Geometric means and 95% confidence intervals of the HIV-1-specific CD4^+^ T-cell responses in macaques. (PDF)

S2 Table Cytokine expression profiles of HIV-1-specific CD4^+^ T-cell responses in individual macaques. (PDF)

S3 Table Geometric means and 95% confidence intervals of the HIV-1-specific CD8^+^ T-cell responses in macaques. (PDF)

S4 Table Cytokine expression profiles of HIV-1-specific CD8^+^ T-cell responses in individual macaques. (PDF)

S5 Table HIV-1 antigens targeted by CD4^+^ and CD8^+^ T-cell responses in individual macaques at 2 weeks post last immunization. (PDF)

S6 Table Phenotype of memory HIV-1-specific T cells CD4^+^ T cells in individual macaques at 6 months post last immunization. (PDF)

S7 Table Phenotype of memory HIV-1-specific T cells CD8^+^ T cells in individual macaques at 6 months post last immunization. (PDF)

S8 Table Humoral responses against the F4 antigen in individual macaques. (PDF)
