# Supplementary material for: Heterologous Prime-Boost Regimens with a Recombinant Chimpanzee Adenoviral Vector and Adjuvanted F4 Protein Elicit Polyfunctional HIV-1-Specific T-Cell Responses in Macaques
Source: PLoS One. 2015 Apr 9;10(4):e0122835. doi: 10.1371/journal.pone.0122835 (PMC4391709; doi:10.1371/journal.pone.0122835)
Supplement: S1 Table — (PDF) [file pone.0122835.s001.pdf]

S1 Table. Geometric means and 95% confidence intervals of the HIV-1-specific $CD4^+$ T-cell responses in macaques

GROUP	Week	Geomean (%)	Lower 95% CI (%)	Upper 95% CI (%)
AA	0	0.00889	0.00254	0.03111
AA	2	0.04727	0.02642	0.08458
AA	14	0.15763	0.10854	0.22892
AA	24	0.08741	0.04753	0.16075
AA	30	0.06477	0.0338	0.12411
AAPP	0	0.0208	0.0068	0.06365
AAPP	2	0.06766	0.03705	0.12358
AAPP	14	0.06635	0.04655	0.09456
AAPP	24	0.05196	0.02405	0.11226
AAPP	28	0.26757	0.15793	0.45335
AAPP	30	0.35748	0.2292	0.55755
AAPP	40	0.16736	0.09945	0.28164
AAPP	52	0.11073	0.05695	0.21529
PP	0	0.02933	0.01385	0.06213
PP	2	0.12599	0.05272	0.30108
PP	6	0.40752	0.24236	0.68523
PP	16	0.30154	0.18555	0.49003
PP	28	0.15136	0.09562	0.23958
PPAA	0	0.0336	0.01435	0.07866
PPAA	2	0.13275	0.07462	0.23617
PPAA	6	0.48092	0.23521	0.98333
PPAA	16	0.19092	0.0752	0.48467
PPAA	18	0.15035	0.01775	1.27382
PPAA	30	0.26909	0.12636	0.57305
PPAA	40	0.18636	0.10588	0.328
PPAA	52	0.10859	0.04415	0.26706

Data relate to those presented in Figure 1A.
