# Supplementary material for: Heterologous Prime-Boost Regimens with a Recombinant Chimpanzee Adenoviral Vector and Adjuvanted F4 Protein Elicit Polyfunctional HIV-1-Specific T-Cell Responses in Macaques
Source: PLoS One. 2015 Apr 9;10(4):e0122835. doi: 10.1371/journal.pone.0122835 (PMC4391709; doi:10.1371/journal.pone.0122835)
Supplement: S2 Table — (PDF) [file pone.0122835.s002.pdf]

S2 Table. Cytokine expression profiles of HIV-1-specific CD4^{+} T-cell responses in individual macaques

		Frequencies per CD4^{+} T-cell phenotype (%)						
**Group**	**Monkey ID no.**	**IL-2^{+} IFN-γ^{-} TNF-α^{-}**	**IL-2^{-} IFN-γ^{+} TNF-α^{-}**	**IL-2^{-} IFN-γ^{-} TNF-α^{+}**	**IL-2^{+} IFN-γ^{+} TNF-α^{-}**	**IL-2^{+} IFN-γ^{-} TNF-α^{+}**	**IL-2^{-} IFN-γ^{+} TNF-α^{+}**	**IL-2^{+} IFN-γ^{+} TNF-α^{+}**
AA	2	0.0317	0.0188	0.0012	0.0388	0	0	0.003
AA	14	0.0381	0.0462	0.0097	0.0817	0.0153	0.0149	0.0461
AA	18	0.011	0.0028	0.0151	0.0108	0.0108	0.0167	0.0224
AA	20	0.014	0.0677	0.0273	0.0656	0.0209	0.0098	0.0634
AA	30	0.0416	0.0202	0.005	0.0308	0.0282	0	0.0211
AA	50	0.0487	0.028	0.0194	0.0647	0.0194	0.0021	0.0324
AA	29	0.0305	0.022	0.0143	0.0244	0.0184	0.0124	0.0169
AA	35	0.0153	0.0536	0	0.0757	0.0051	0.0077	0.0452
PP	7	0.217	0.008	0.003	0.022	0.052	0	0.011
PP	9	0.651	0.024	0.037	0.07	0.16	0.015	0.009
PP	3	0.211	0	0.022	0.024	0.149	0	0.028
PP	22	0.74	0	0.015	0.033	0.293	0	0.012
PP	45	0.145	0.019	0.014	0.046	0.027	0.009	0.019
PP	47	0.226	0	0.02	0.017	0.102	0.014	0.014
PP	28	0.145	0.019	0.014	0.046	0.027	0.009	0.019
PP	31	0.226	0	0.02	0.017	0.102	0.014	0.014
PPAA	6	0.021	0.016	0	0.033	0.002	0.005	0.005
PPAA	8	0	0	0	0.008	0.025	0.035	0.076
PPAA	23	0.045	0.051	0.017	0.048	0.041	0.004	0.028
PPAA	25	0.075	0.029	0.019	0.037	0.038	0.003	0.032
PPAA	43	0.333	0.101	0.069	0.147	0.226	0.019	0.117
PPAA	27	0.08	0.079	0.012	0.109	0.071	0.018	0.115
PPAA	32	0.081	0.06	0.028	0.059	0.043	0.011	0.043
AAPP	4	0.02	0.046	0	0.06	0.007	0	0.046
AAPP	11	0.046	0.19	0	0.171	0.007	0.013	0.054
AAPP	5	0.052	0.161	0.008	0.183	0.013	0.017	0.062
AAPP	19	0.024	0.052	0.006	0.071	0.017	0.01	0.009
AAPP	37	0.094	0.166	0.019	0.091	0.036	0.006	0.049
AAPP	46	0.055	0.047	0.007	0.096	0.004	0.013	0.048
AAPP	26	0.158	0.331	0.022	0.233	0.025	0.021	0.057
AAPP	38	0.075	0.156	0.015	0.033	0.009	0.016	0.009

Data relate to those presented in Figure 1B.
