# Supplementary material for: Heterologous Prime-Boost Regimens with a Recombinant Chimpanzee Adenoviral Vector and Adjuvanted F4 Protein Elicit Polyfunctional HIV-1-Specific T-Cell Responses in Macaques
Source: PLoS One. 2015 Apr 9;10(4):e0122835. doi: 10.1371/journal.pone.0122835 (PMC4391709; doi:10.1371/journal.pone.0122835)
Supplement: S3 Table — (PDF) [file pone.0122835.s003.pdf]

S3 Table. Geometric means and 95% confidence intervals of the HIV-1-specific $CD8^+$ T-cell responses in macaques

Group	Week	Geomean (%)	Lower 95% CI (%)	Upper 95% CI (%)
AA	0	0.01934	0.0055	0.06794
AA	2	0.17462	0.0655	0.4655
AA	14	0.51439	0.25519	1.03687
AA	24	0.2454	0.12499	0.48183
AA	30	0.12134	0.06328	0.23267
AAPP	0	0.02324	0.0064	0.08445
AAPP	2	0.1971	0.11539	0.33665
AAPP	14	0.32537	0.15411	0.68694
AAPP	24	0.18217	0.09748	0.34042
AAPP	28	0.16364	0.0779	0.34376
AAPP	30	0.17403	0.10753	0.28166
AAPP	40	0.11017	0.05985	0.20282
AAPP	52	0.0628	0.035	0.11268
PP	0	0.0501	0.02872	0.0874
PP	2	0.02753	0.01055	0.07182
PP	6	0.04592	0.02869	0.0735
PP	16	0.02262	0.00387	0.13208
PP	28	0.02585	0.0106	0.06302
PPAA	0	0.04447	0.01548	0.12778
PPAA	2	0.04218	0.01451	0.12262
PPAA	6	0.0391	0.01497	0.10211
PPAA	16	0.03329	0.01348	0.0822
PPAA	18	0.11181	0.04471	0.27958
PPAA	30	0.43366	0.14476	1.29913
PPAA	40	0.13914	0.04308	0.44932
PPAA	52	0.09524	0.02856	0.3176

Data relate to those presented in Figure 2A.
