# Supplementary material for: Heterologous Prime-Boost Regimens with a Recombinant Chimpanzee Adenoviral Vector and Adjuvanted F4 Protein Elicit Polyfunctional HIV-1-Specific T-Cell Responses in Macaques
Source: PLoS One. 2015 Apr 9;10(4):e0122835. doi: 10.1371/journal.pone.0122835 (PMC4391709; doi:10.1371/journal.pone.0122835)
Supplement: S4 Table — (PDF) [file pone.0122835.s004.pdf]

S4 Table. Cytokine expression profiles of HIV-1-specific CD8$^+$ T-cell responses in individual macaques

		Frequencies per CD8$^+$ T-cell phenotype (%)						
**Groupe**	**Monkey ID**	**IL-2$^+$ IFN-γ$^-$ TNF-α$^-$**	**IL-2$^-$ IFN-γ$^+$ TNF-α$^-$**	**IL-2$^-$ IFN-γ$^-$ TNF-α$^+$**	**IL-2$^+$ IFN-γ$^+$ TNF-α$^-$**	**IL-2$^+$ IFN-γ$^-$ TNF-α$^+$**	**IL-2$^-$ IFN-γ$^+$ TNF-α$^+$**	**IL-2$^+$ IFN-γ$^+$ TNF-α$^+$**
AA	2	0.011276071	0.161907931	0.036083856	0.046847184	0.007722753	0.217297088	0.109587234
AA	14	0	0.216217899	0.030866414	0.004118276	0	0.124701772	0.028765305
AA	18	0.005151831	0.23178175	0.001052686	0.018492834	0.014952153	0.330392945	0.042137499
AA	20	0.007806401	0.21614895	0.016047275	0	0	0.310104036	0.007821666
AA	30	0.001596372	0.026521603	0.001186012	0.005068424	0.002658443	0.038566114	0.006247908
AA	50	0	0.411696669	0.036912227	0.00923386	0.004605324	0.274644799	0.054075142
AA	29	0.026254111	0.340803997	0	0.027828546	0	0.174668096	0
AA	35	0.010473645	0.810145009	0.111783531	0.05223851	0.014128826	0.519462726	0.029204752
PP	7	0.009592326	0.032326543	0	0.009592326	0	0.004796163	0
PP	9	0.008770391	0.015278546	0.026447116	0.003922531	0	0.004385196	0.004385196
PP	3	0.006438981	0.006438981	0.005674081	0	0	0.0026758	0.002350219
PP	22	0.040468773	0.022166527	0.022211673	0.004589051	0.002294525	3.30E-05	0.002294525
PP	45	0.001152312	0.009670765	0	0.003773015	0	0	0.003773015
PP	47	0	0.0328526	0	0.003725505	0	0	0
PP	28	0	0.018504052	0.031183659	0.001602073	0	0	0
PP	31	0.00339075	0.016867421	0	0	0.005788377	0.039279134	0.007359216
PPAA	6	0.008595496	0.824460004	0.020569557	0.017190992	0.008595496	0.152210964	0.008595496
PPAA	8	0	0.202519823	0.013218031	0	0.001015667	0.192348792	0
PPAA	23	0	0.060570521	0.019184352	0.002888737	0.008360305	0.041594499	0.01907213
PPAA	25	0.012159083	0.030963076	0.003529858	0.028459229	0	0.012545032	0.004787722
PPAA	43	0.039892347	1.129232786	0.069540605	0.228314773	0.02418692	0.351551976	0.312232581
PPAA	27	0.00453114	0.757442366	0.013630096	0.03968991	0.005238894	0.267998769	0.068930683
PPAA	32	0.002782105	0.144959321	0	0.032947187	0	0.00554927	0.007574167
AAPP	4	0.010191083	0.13561236	0	0.027350014	0	0.086730811	0.045859873
AAPP	11	0	0.143906893	0	0.047949087	0	0.113483286	0.065385118
AAPP	5	0.002022156	0.174012014	0.008088623	0.034410681	0.008409419	0.067160538	0.034779683
AAPP	19	0.000430449	0	0.036295262	0.00048493	0.002877367	0.038470908	0.00842075
AAPP	37	0	0.042973786	0.043205253	0.012278225	0	0.043031653	0
AAPP	46	0.007181821	0.061334245	0	0.037487056	0.003464283	0.018954494	0.007181821
AAPP	26	0.011746042	0.043801526	0.01916234	0	0	0.058240994	0.015042352
AAPP	38	0.00805419	0.016899107	0.003433061	0.007829168	0	0.052383579	0.002789712

Data relate to those presented in Figure 2B.
