# Supplementary material for: Heterologous Prime-Boost Regimens with a Recombinant Chimpanzee Adenoviral Vector and Adjuvanted F4 Protein Elicit Polyfunctional HIV-1-Specific T-Cell Responses in Macaques
Source: PLoS One. 2015 Apr 9;10(4):e0122835. doi: 10.1371/journal.pone.0122835 (PMC4391709; doi:10.1371/journal.pone.0122835)
Supplement: S5 Table — (PDF) [file pone.0122835.s005.pdf]

S5 Table. HIV-1 antigens targeted by $CD4^+$ and $CD8^+$ T-cell responses in individual macaques at 2 weeks post last immunization

Group	Monkey ID	Stimulation	$CD4^+$ T cells (%)	$CD8^+$ T cells (%)
AA	2	Nef	0.11703	0.207544331
AA	2	P17	0.03775	0.084387352
AA	2	P24	0.06253	0.040762626
AA	2	RT	0.10840	0.428585014
AA	14	Nef	0.04141	0.200626705
AA	14	P17	0.03766	0.064477864
AA	14	P24	0.03803	0.004179454
AA	14	RT	0.15065	0.256598825
AA	18	Nef	0.08747	0
AA	18	P17	0.06310	0.040063773
AA	18	P24	0.04140	0.106747099
AA	18	RT	0.14974	0.679635407
AA	20	Nef	0.01913	0.023573152
AA	20	P17	0.04449	0.066263552
AA	20	P24	0.07108	0.022563177
AA	20	RT	0.03183	0.508304854
AA	29	Nef	0.04785	0.067579239
AA	29	P17	0.04815	0.064438085
AA	29	P24	0.11628	0.458740674
AA	29	RT	0.10369	0.57342916
AA	30	Nef	0.07685	0.030792936
AA	30	P17	0.01891	0.03252548
AA	30	P24	0.07017	0.03215072
AA	30	RT	0.10487	0.082143495
AA	35	Nef	0.03206	0.045569765
AA	35	P17	0.03563	0.08604539
AA	35	P24	0.04389	0.158911903
AA	35	RT	0.08003	1.39011396
AA	50	Nef	0.05222	0.10180572
AA	50	P17	0.03980	0.025046447
AA	50	P24	0.05414	0.164251786
AA	50	RT	0.05441	0.225561891
PP	3	Nef	0.10952	0.024319473
PP	3	P17	0.13705	0.012847842
PP	3	P24	0.18803	0.031530476
PP	3	RT	0.22564	0.036511224
PP	7	Nef	0.01987	0.040078486
PP	7	P17	0.03349	0.018818212
PP	7	P24	0.10423	0.026766232
PP	7	RT	0.06205	0.038385312
PP	9	Nef	0.16364	0.057700539
PP	9	P17	0.13463	0.061465461
PP	9	P24	0.29834	0.060523596
PP	9	RT	0.41211	0.047737625
PP	22	Nef	0.16512	0.11562704
PP	22	P17	0.07990	0.138150638

Group	Monkey ID	Stimulation	CD4$^+$ T cells (%)	CD8$^+$ T cells (%)
PP	22	P24	0.34592	0.062051182
PP	22	RT	0.69501	0.066600938
PP	28	Nef	0.10027	0.066759509
PP	28	P17	0.06782	0.109465699
PP	28	P24	0.18584	0.047509806
PP	28	RT	0.23600	0.060469297
PP	31	Nef	0.01664	0.107124522
PP	31	P17	0.00716	0.05645492
PP	31	P24	0.02214	0.04453301
PP	31	RT	0.11829	0.153718711
PP	45	Nef	0.08644	0.100378322
PP	45	P17	0.04239	0.022407947
PP	45	P24	0.15119	0.094640705
PP	45	RT	0.20014	0.006129642
PP	47	Nef	0.08732	0.040459835
PP	47	P17	0.06648	0.043703969
PP	47	P24	0.02483	0.059242227
PP	47	RT	0.35316	0.05325612
PPAA	6	Nef	0.02584	0.05982463
PPAA	6	P17	0.00205	0.088315252
PPAA	6	P24	0.04140	0.09499487
PPAA	6	RT	0.03148	1.009013947
PPAA	8	Nef	0.03346	0.033750418
PPAA	8	P17	0.01809	0.046224681
PPAA	8	P24	0.02094	0.084744597
PPAA	8	RT	0.28522	0.263624921
PPAA	23	Nef	0.03176	0.047378192
PPAA	23	P17	0.01690	0.026922025
PPAA	23	P24	0.03235	0.077136676
PPAA	23	RT	0.14576	0.049470116
PPAA	25	Nef	0.03801	0.020827638
PPAA	25	P17	0.01030	0.020425217
PPAA	25	P24	0.04014	0.008270028
PPAA	25	RT	0.19740	0.074672567
PPAA	27	Nef	0.04388	0.888863201
PPAA	27	P17	0.04048	0.034854546
PPAA	27	P24	0.05757	0.196576945
PPAA	27	RT	0.37547	0.06445538
PPAA	32	Nef	0.06721	0.109340593
PPAA	32	P17	0.04912	0.080651724
PPAA	32	P24	0.05402	0.044137196
PPAA	32	RT	0.24321	0.190504271
PPAA	42	Nef	#N/A	#N/A
PPAA	42	P17	#N/A	#N/A
PPAA	42	P24	#N/A	#N/A
PPAA	42	RT	#N/A	#N/A
PPAA	43	Nef	0.27482	0.217722302
PPAA	43	P17	0.07257	0.55068248
PPAA	43	P24	0.09386	0.047583483

Group	Monkey ID	Stimulation	CD4^{+} T cells (%)	CD8^{+} T cells (%)
PPAA	43	RT	0.59775	0.726487835
AAPP	4	Nef	0.04593	0.214139273
AAPP	4	P17	0.00050	0.116405405
AAPP	4	P24	0.01726	0.136531192
AAPP	4	RT	0.07188	0.286162635
AAPP	5	Nef	0.05043	0.012268755
AAPP	5	P17	0.08544	0.01053013
AAPP	5	P24	0.11632	0.03396072
AAPP	5	RT	0.37287	0.29747013
AAPP	11	Nef	0.01872	0.165927997
AAPP	11	P17	0.12731	0.095499404
AAPP	11	P24	0.16384	0.013197629
AAPP	11	RT	0.24956	0.079551966
AAPP	19	Nef	0.01921	0.071275011
AAPP	19	P17	0.04248	0.078495174
AAPP	19	P24	0.02667	0.059934302
AAPP	19	RT	0.10093	0.071317769
AAPP	26	Nef	0.12505	0.06638162
AAPP	26	P17	0.04054	0.042968612
AAPP	26	P24	0.10970	0.085281787
AAPP	26	RT	0.56583	0.066471356
AAPP	37	Nef	0.04483	0.042809148
AAPP	37	P17	0.08220	0.061393038
AAPP	37	P24	0.09615	0.07325863
AAPP	37	RT	0.12296	0.099910263
AAPP	38	Nef	0.05088	0.030905398
AAPP	38	P17	0.05808	0.038429069
AAPP	38	P24	0.31578	0.053021438
AAPP	38	RT	0.15383	0.072893612
AAPP	46	Nef	0.04263	0.007662888
AAPP	46	P17	0.01809	0.02093344
AAPP	46	P24	0.09339	0.005644137
AAPP	46	RT	0.22992	0.107924793

Data relate to those presented in Figure 3.
