# Supplementary material for: Heterologous Prime-Boost Regimens with a Recombinant Chimpanzee Adenoviral Vector and Adjuvanted F4 Protein Elicit Polyfunctional HIV-1-Specific T-Cell Responses in Macaques
Source: PLoS One. 2015 Apr 9;10(4):e0122835. doi: 10.1371/journal.pone.0122835 (PMC4391709; doi:10.1371/journal.pone.0122835)
Supplement: S6 Table — (PDF) [file pone.0122835.s006.pdf]

S6 Table. Phenotype of memory HIV-1-specific T cells CD4$^+$ T cells in individual macaques at 6 months post last immunization

					Frequency		
Group	Monkey ID no.	Timing	Stimulation	Memory population	% IL-2$^+$	% INF-γ$^+$	% INF-γ$^+$ IL-2$^+$
AA	35	180dpIV	Pool F4	EM	0.024	0.000	0.000
				CM	0.024	0.165	0.118
AA	2	180dpIV	Pool F4	EM	0.000	0.000	0.000
				CM	0.013	0.027	0.112
AA	50	180dpII	Pool F4	EM	0.000	0.000	0.000
				CM	0.000	0.042	0.036
AA	29	180dpII	Pool F4	EM	0.000	0.000	0.000
				CM	0.083	0.000	0.179
AA	18	180dpII	Pool F4	EM	0.036	0.038	0.077
				CM	0.000	0.150	0.008
AA	20	180dpII	Pool F4	EM	0.000	0.000	0.000
				CM	0.010	0.060	0.114
AA	30	180dpII	Pool F4	EM	0.000	0.000	0.000
				CM	0.000	0.035	0.083
AA	14	180dpII	Pool F4	EM	0.011	0.000	0.000
				CM	0.022	0.021	0.034
PPAA	43	180dpIV	Pool F4	EM	0.015	0.000	0.000
				CM	0.031	0.000	0.124
PPAA	27	180dpIV	Pool F4	EM	0.000	0.000	0.000
				CM	0.081	0.024	0.118
PPAA	25	180dpIV	Pool F4	EM	0.000	0.029	0.000
				CM	0.000	0.000	0.000
PPAA	23	180dpII	Pool F4	EM	0.000	0.000	0.000
				CM	0.090	0.177	0.206
PPAA	8	180dpII	Pool F4	EM	0.000	0.163	0.000
				CM	0.009	0.018	0.009
PPAA	6	180dpII	Pool F4	EM	0.000	0.008	0.004
				CM	0.024	0.158	0.103
PPAA	32	180dpII	Pool F4	EM	0.000	0.016	0.016
				CM	0.034	0.086	0.032
AAPP	37	180dpIV	Pool F4	EM	0.000	0.000	0.000
				CM	0.000	0.000	0.023
AAPP	38	180dpIV	Pool F4	EM	0.011	0.000	0.000
				CM	0.045	0.018	0.029
AAPP	26	180dpIV	Pool F4	EM	0.000	0.000	0.000
				CM	0.079	0.126	0.084
AAPP	4	180dpII	Pool F4	EM	0.000	0.023	0.000
				CM	0.000	0.091	0.000
AAPP	11	180dpII	Pool F4	EM	0.000	0.000	0.009
				CM	0.036	0.288	0.099
AAPP	5	180dpII	Pool F4	EM	0.000	0.000	0.000
				CM	0.008	0.224	0.187
AAPP	19	180dpII	Pool F4	EM	0.000	0.030	0.000
				CM	0.030	0.181	0.010
AAPP	46	180dpII	Pool F4	EM	0.000	0.000	0.000
				CM	0.029	0.096	0.058
PP	31	180dpIV	Pool F4	EM	0.000	0.000	0.000
				CM	0.274	0.000	0.012
PP	28	180dpIV	Pool F4	EM	0.023	0.000	0.000
				CM	0.000	0.000	0.000
PP	22	180dpII	Pool F4	EM	0.000	0.000	0.000
				CM	0.978	0.031	0.061
PP	3	180dpII	Pool F4	EM	0.000	0.000	0.000
				CM	0.336	0.012	0.037
PP	9	180dpII	Pool F4	EM	0.000	0.000	0.000
				CM	0.108	0.000	0.077
PP	7	180dpII	Pool F4	EM	0.017	0.008	0.000
				CM	0.191	0.035	0.026
PP	45	180dpII	Pool F4	EM	0.008	0.000	0.000
				CM	0.089	0.033	0.097
PP	47	180dpII	Pool F4	EM	0.000	0.000	0.000
				CM	0.112	0.000	0.007

Data relate to those presented in Figure 4A.
