# Supplementary material for: Heterologous Prime-Boost Regimens with a Recombinant Chimpanzee Adenoviral Vector and Adjuvanted F4 Protein Elicit Polyfunctional HIV-1-Specific T-Cell Responses in Macaques
Source: PLoS One. 2015 Apr 9;10(4):e0122835. doi: 10.1371/journal.pone.0122835 (PMC4391709; doi:10.1371/journal.pone.0122835)
Supplement: S7 Table — (PDF) [file pone.0122835.s007.pdf]

S7 Table. Phenotype of memory HIV-1-specific T cells CD8$^+$ T cells in individual macaques at 6 months post last immunization

					Frequency		
Group	Monkey ID no.	Timing	Stimulation	Memory population	% IL-2$^+$	% INF-γ$^+$	% INF-γ$^+$ IL-2$^+$
AA	35	180dpIV	Pool F4	EM	0.000	0.026	0.000
				CM	0.020	0.054	0.014
AA	2	180dpIV	Pool F4	EM	0.020	0.085	0.000
				CM	0.011	0.265	0.080
AA	50	180dpII	Pool F4	EM	0.000	0.040	0.000
				CM	0.000	0.063	0.023
AA	29	180dpII	Pool F4	EM	0.000	0.093	0.000
				CM	0.000	0.021	0.000
AA	18	180dpII	Pool F4	EM	0.054	0.161	0.011
				CM	0.053	0.064	0.011
AA	20	180dpII	Pool F4	EM	0.022	0.043	0.011
				CM	0.000	0.078	0.000
AA	30	180dpII	Pool F4	EM	0.000	0.000	0.000
				CM	0.000	0.019	0.000
AA	14	180dpII	Pool F4	EM	0.000	0.132	0.006
				CM	0.000	0.088	0.019
PPAA	43	180dpIV	Pool F4	EM	0.000	0.169	0.011
				CM	0.000	0.358	0.011
PPAA	27	180dpIV	Pool F4	EM	0.000	0.362	0.035
				CM	0.000	0.219	0.023
PPAA	25	180dpIV	Pool F4	EM	0.000	0.002	0.000
				CM	0.000	0.000	0.000
PPAA	23	180dpII	Pool F4	EM	0.038	0.000	0.000
				CM	0.000	0.000	0.046
PPAA	8	180dpII	Pool F4	EM	0.000	0.048	0.000
				CM	0.000	0.005	0.011
PPAA	6	180dpII	Pool F4	EM	0.033	0.068	0.070
				CM	0.000	0.000	0.000
PPAA	32	180dpII	Pool F4	EM	0.010	0.000	0.003
				CM	0.023	0.332	0.060
AAPP	37	180dpIV	Pool F4	EM	0.017	0.077	0.000
				CM	0.000	0.000	0.000
AAPP	38	180dpIV	Pool F4	EM	0.011	0.149	0.011
				CM	0.011	0.124	0.022
AAPP	26	180dpIV	Pool F4	EM	0.010	0.000	0.000
				CM	0.011	0.042	0.000
AAPP	4	180dpII	Pool F4	EM	0.000	0.000	0.000
				CM	0.000	0.000	0.021
AAPP	11	180dpII	Pool F4	EM	0.000	0.059	0.013
				CM	0.000	0.093	0.073
AAPP	5	180dpII	Pool F4	EM	0.000	0.096	0.000
				CM	0.000	0.056	0.016
AAPP	19	180dpII	Pool F4	EM	0.023	0.000	0.007
				CM	0.047	0.050	0.016
AAPP	46	180dpII	Pool F4	EM	0.027	0.005	0.007
				CM	0.126	0.021	0.038
PP	31	180dpIV	Pool F4	EM	0.000	0.000	0.012
				CM	0.002	0.000	0.000
PP	28	180dpIV	Pool F4	EM	0.000	0.033	0.000
				CM	0.000	0.000	0.000
PP	22	180dpII	Pool F4	EM	0.000	0.000	0.000
				CM	0.006	0.000	0.000
PP	3	180dpII	Pool F4	EM	0.000	0.000	0.007
				CM	0.000	0.000	0.000
PP	9	180dpII	Pool F4	EM	0.002	0.016	0.006
				CM	0.006	0.005	0.000
PP	7	180dpII	Pool F4	EM	0.001	0.000	0.018
				CM	0.027	0.000	0.009
PP	45	180dpII	Pool F4	EM	0.044	0.000	0.000
				CM	0.000	0.044	0.000
PP	47	180dpII	Pool F4	EM	0.000	0.000	0.000
				CM	0.000	0.000	0.000

Data relate to those presented in Figure 4B.
