# Supplementary material for: Heterologous Prime-Boost Regimens with a Recombinant Chimpanzee Adenoviral Vector and Adjuvanted F4 Protein Elicit Polyfunctional HIV-1-Specific T-Cell Responses in Macaques
Source: PLoS One. 2015 Apr 9;10(4):e0122835. doi: 10.1371/journal.pone.0122835 (PMC4391709; doi:10.1371/journal.pone.0122835)
Supplement: S8 Table — (PDF) [file pone.0122835.s008.pdf]

S8 Table. Humoral responses against the F4 antigen in individual macaques

		Titer				
**Group**	**Monkey ID no.**	**Pre**	**14/16 pI**	**13pII**	**83pII**	**167pII**
AA	2	500	500	9203	615	770
AA	14	500	500	18886	828	1079
AA	18	500	500	11238	500	500
AA	20	500	500	500	500	500
AA	30	500	500	12758	1097	1325
AA	50	500	500	11116	827	500
AA	29	500	500	11322	1102	947
AA	35	500	500	6016	500	500

Group	Monkey ID no.	Pre	14/16 pI	14pII	84pII	168pII
PP	7	500	500	24811	1059	1685
PP	9	500	2761	58460	4728	6499
PP	3	500	500	44164	1434	1658
PP	22	500	500	54921	3100	3215
PP	45	500	1362	52019	4930	5921
PP	47	500	500	15597	1853	1328
PP	28	500	920	27901	2825	3773
PP	31	500	500	6610	519	500

Group	Monkey ID no.	Pre	14/16 pI	13pII	83pII	13pIII	13pIV	84pIV	197pIV
AAPP	4	500	500	2911	500	52224	73994	2830	1326
AAPP	11	500	500	5602	500	103670	64322	4502	2450
AAPP	5	500	500	18620	500	71004	70431	5403	2906
AAPP	19	500	500	6027	603	40995	47181	2223	1270
AAPP	37	500	500	9528	804	31800	54919	5880	3545
AAPP	46	500	500	19777	679	45765	35311	2481	1483
AAPP	26	500	500	3396	500	66485	69515	4462	5759
AAPP	38	500	500	5682	1825	53672	63909	4156	2398

Group	Monkey ID no.	Pre	14/16 pI	14pII	84pII	13pIII	13pIV	82pIV	167pIV
PPAA	6	500	2024	86436	7641	68115	23965	11199	9369
PPAA	8	500	500	40159	3991	62606	19525	9524	8550
PPAA	23	500	1756	75747	7229	161625	46610	20371	23157
PPAA	25	500	1264	80518	7820	122323	51392	14964	10210
PPAA	42	500	1003	64841	#N/A	#N/A	#N/A	#N/A	#N/A
PPAA	43	500	729	39534	3292	47333	55030	15641	11230
PPAA	27	500	644	29438	2164	112262	43220	14453	8438
PPAA	32	500	4551	118867	13798	96099	26544	11692	7918

Data relate to those presented in Figure 5.
